# Correction: The burden of respiratory conditions in the emergency department of Muhimbili National Hospital in Tanzania in the first two years of the COVID-19 pandemic: A cross sectional descriptive study

**DOI:** 10.1371/journal.pgph.0002125

**Published:** 2023-06-26

**Authors:** Harrieth P. Ndumwa, Erick A Mboya, Davis Elias Amani, Ramadhani Mashoka, Paulina Nicholaus, Rashan Haniffa, Abi Beane, Juma Mfinanga, Bruno Sunguya, Hendry R. Sawe, Tim Baker

There are errors in the Funding statement. The correct Funding statement is as follows: This study was supported by the African critical care registry network for pandemic surveillance, clinical management and research (MRC/UKRI MR/V030884/1), awarded to RH, AB and TB. This study was also supported by the Laerdal Foundation (2021–0097), awarded to TB, HPN. This study was also funded in whole, or in part, by the Wellcome Trust (220211), awarded to RH, AB and TB. For the purpose of Open Access, the author has applied a CC BY public copyright license to any Author Accepted Manuscript version arising from this submission. HPN, EAM, RM and PN received salary support from the African critical care registry network for pandemic surveillance, clinical management and research funding for coordination and technical activities of the project. The specific roles of these authors are articulated in the ‘author contributions’ section. The funders had no role in study design, data collection and analysis, decision to publish, or preparation of the manuscript.
