# Supplementary material for: The burden of respiratory conditions in the emergency department of Muhimbili National Hospital in Tanzania in the first two years of the COVID-19 pandemic: A cross sectional descriptive study
Source: PLOS Glob Public Health. 2022 Dec 9;2(12):e0000781. doi: 10.1371/journal.pgph.0000781 (PMC10021642; doi:10.1371/journal.pgph.0000781)
Supplement: Attachment — Submitted filename: Response to reviewers.docx [file pgph.0000781.s001.docx]

Dear Editor,

Thank you for taking the time to consider our manuscript titled “**The burden of respiratory conditions in the emergency department of Muhimbili National Hospital in Tanzania in the first two years of the COVID-19 pandemic”**-PGPH-D-22-01035. We have made revisions in the manuscript to address the comments from you and from the reviewers and it has improved as a result. The revised manuscript is attached in a tracked-changes version and a clean version. We have also responded point-by-point to the reviewers’ comments below.

Yours sincerely,

Harrieth P Ndumwa

On behalf of the authors

| **Editor(s)' Comments to the Authors** | **Our response** |
| --- | --- |
| 1. We noticed that you have cited Figures 1-7 in the manuscript. However, there are no regular figures 1-7 but only supplementary figures. Please update the citations/labels to matched with the uploaded files. | Thank you for the comment. We have updated the labels to match with the uploaded files. |
| 2. In the online submission form, you indicated that "Data can be made available upon reasonable request from the corresponding author. “All PLOS journals now require all data underlying the findings described in their manuscript to be freely available to other researchers, either 1. In a public repository, 2. Within the manuscript itself, or 3. Uploaded as supplementary information.   This policy applies to all data except where public deposition would breach compliance with the protocol approved by your research ethics board. If your data cannot be made publicly available for ethical or legal reasons (e.g., public availability would compromise patient privacy), please explain your reasons by return email and your exemption request will be escalated to the editor for approval. Your exemption request will be handled independently and will not hold up the peer review process, but will need to be resolved should your manuscript be accepted for publication. One of the Editorial team will then be in touch if there are any issues. | Thank you for this comment.  For ethical and legal reasons in Tanzania, data used in this manuscript cannot be made publicly available, however, upon request and following national policies in data sharing, these can be supplied to any researcher requesting and fulfilling requirements as per the Tanzanian National Health Research Ethics guidelines.  Additionally, patients did not consent to have their raw data shared in public servers or in any journal’s servers.  We are therefore requesting an exemption on this.  The data availability statement section in the manuscript has been amended accordingly. |
| 3. Please update the 'Competing Interests' statement, including any COIs declared by your co-authors. If you have no competing interests to declare, please state "The authors have declared that no competing interests exist". Otherwise please declare all competing interests beginning with the statement "I have read the journal's policy and the authors of this manuscript have the following competing interests:" | Thank you.  The competing interests statement has been updated. |
| 4. Please amend your detailed Financial Disclosure statement. This is published with the article. It must therefore be completed in full sentences and contain the exact wording you wish to be published. a. Please clarify all sources of funding (financial or material support) for your study. List the grants (with grant number) or organizations (with url) that supported your study, including funding received from your institution. b. State the initials, alongside each funding source, of each author to receive each grant. c. State what role the funders took in the study. If the funders had no role in your study, please state: “The funders had no role in study design, data collection and analysis, decision to publish, or preparation of the manuscript.” d. If any authors received a salary from any of your funders, please state which authors and which funders. | Thank you for the comment. We have amended the financial disclosure statement as instructed. |

| **Reviewer 1 comments** | **Our response** |
| --- | --- |
| I want to commend the researchers in their efforts to contribute to scientific knowledge. | Thank you! |
| From my review, I believe the work is original. I can’t comment on whether it has been published elsewhere. this is an observational study, hence, no experiments were carried out. I do feel that the conclusion need to be revisited, as it states that 52% of the total respiratory cases seen were due to pneumonia while 2.5% were due to COVID-19 [which is also a form of pneumonia]. the paper did not state how it differentiated COVID-19 pneumonia from other causes of pneumonia. the authors wrote that testing capacity was limited but did not mention the use of case definitions which can be used to identify cases in an epidemic/pandemic. | Thank you for this very important comment.  In addition to demonstrating the high burden of respiratory illness during the pandemic, the key finding from the study is the use of hospital-based surveillance tools in early detection of pandemics and other disease outbreaks in similar settings where diagnostic tests are limited.  Note that the diagnoses were made by the clinical teams in the emergency unit and not by the researchers. All laboratory results such as COVID-19 were not always available and clinicians relied on the history, clinical signs and available investigation results to make preliminary diagnoses..  The methods section and the conclusion have been revised to accommodate the proposed comments and hence further clarity. |
| I recommend the research title include the design according to the STROBE checklist for manuscript writing | Thank you for the comment, the study design has been captured in the manuscript title. |
| I also recommend that the limitations of the study be exhaustively stated. | Thank you!  The limitations have been expounded. |
